# Microscopic Observations Respecting Arterial and Capillary Circulation

**Published:** 1851-12

**Authors:** J. H. Wythes

**Affiliations:** Paoli, Chester Co., Pennsylvania


*Microscopic Observations respecting Arterial and Capillary Circulation.* By J. H. WYTHES, M. D., of Paoli, Chester Co., Pennsylvania.

It will be readily acknowledged by most physiologists, that the movement of the blood in the capillaries, is to a great extent independent of the action of the heart, since it may continue after the cessation of the heart's action is affected by causes originating in the capillaries themselves, and is present in the vascular area before the development of the heart. The capillary vessels, however, exhibit no obvious movement when examined by the microscope; the blood passing through them in a continuous stream. Now as the only method in which the capillaries could influence the current of blood, is by a peristaltic or pulsatory motion, and as such motion is not observable in them, it seems probable either that their influence has been over-rated, or that the cause and manner of their operation is yet undiscovered.

The arteries, on the contrary, are known to possess both elasticity and contractility. The former of these properties is generally considered to be of a purely physical character, serving to convert the intermittent impulses the blood receives from the heart into a continuous current. The contractility of the middle arterial coat is thought to be a vital property, similar to muscular contractility. A modification of this force is termed tonicity, an example of which is seen in the arrest of hemorrhage by the contraction ensuing on the division of an artery.

The pulsations of the arteries, however, have been regarded as caused by the alternate contraction and dilatation of the heart, and to be equalizing and retarding, rather than propulsive, in their influence on the vital current. Yet physiologists have been inclined to attribute some propulsive influence, supplementary to the heart's action, to the arterial coats. Dr. Carpenter remarks, "If the fibrous coat of the arteries is in some degree disposed to the alternate contraction and relaxation which are so remarkable in the heart, they *may* exert a force which shall be supplementary to that of the heart's impulse, relaxing to receive the blood from it, and contracting upon their contents, with a power superior to that by which they were distended. It is difficult to say whether or not this be the case, though there would certainly appear some evidence in favor of the supposition. The loss of the heart's power over the currents of blood, in proportion to their degree of subdivision, occasioned by the increased friction to which they will be subjected, would seem to require some compensating power, in order that the perfect equality of pressure may be obtained which has been spoken of as existing in all parts of the arterial system. In no other way than this can the fibrous coat of the arteries be regarded as having any propulsive power over their contents, except by a peristaltic or vermicular movement, resembling that which takes place in the alimentary canal; and of such there is no evidence whatever."

It is evident that Dr. Carpenter regards the contraction of an artery upon its contents, to be owing to the stimulus afforded by its distension with blood, which being expended, the vessel is ready to dilate to receive a new supply. The microscopic observation to which we are about to refer, leads the writer of this article to entertain a different view. It seems to be demonstrated by this that the pulsatory movement is a property residing in the arterial coats themselves, independent alike of the heart's action and the stimulus of the blood.

Having caught a mouse in a trap, (it was quite cold and stiff when taken out,) I was desirous of making some preparations of epithelium, &c. On taking out the kidneys it occurred to me to place a thin slice upon a slide for microscopic examination. The slice was made quite through the middle of the kidney, and was about one-thirtieth of an inch in thickness, just thick enough to be translucent. On placing it under the microscope, one of the largest vessels was observed in active motion, alternately contracting and dilating, with evident vermicular contortions, communicating motion to the blood corpuscles in the capillaries for a considerable distance. The movement seemed limited to the artery, and was not communicated to the coats of the capillaries, although their contents had an oscillatory motion corresponding to the contents of the artery. The phenomenon was seen for about three hours, when the observation was suspended. The motion had then considerably diminished both in extent and energy.

I was at first inclined to attribute this activity to evaporation of the watery particles from the slide, but this is insufficient to account for the regular pulsatory character of the movement. It is therefore due, in all probability, to the vital pulsations of the coats of the artery. I have not had an opportunity since that time to repeat the experiment.

The only parallel case with which I am acquainted, is recorded in Hassall's Microscopic Anatomy, as follows:—"On one occasion, in examining the tongue of a frog, a portion of it broke away from the remainder; this I placed between two plates of glass, and submitted to examination, when, extraordinary to say, it was perceived that the circulation was still vigorously maintained in the majority of the vessels. Anxious to know how long this circulation would be continued, but fully expecting to see it cease every moment, myself and a friend, John Coppin, Esq., of Lincoln's Inn, watched it for upwards of an hour, at the end of which time the blood still flowed onwards in many of the vessels, with scarcely abated vigour, though in others, often the larger ones, the motion had altogether ceased. The mutilated portion of the tongue was then placed in water, in which it remained during the whole of the night; the next morning it was again examined, when it was found that a tolerably active circulation still existed in several of the smaller vessels. After this observation, the further examination of the fragment was abandoned."

These observations show:—1. That the pulsation of the arteries is a property residing in the coats of those vessels, which is independent of the heart's action, though supplementary to it; and also independent of the stimulus arising from distension with blood.

2. That a peculiar propulsive force, in all probability, resides in the capillary vessels, of whose nature we are at present uninformed.

3. That one of the chief causes of the capillary circulation, is probably the pulsation of the arterial branches from which they spring.

